# Excitability of Neural Activity is Enhanced, but Neural Discrimination of Odors is Slightly Decreased, in the Olfactory Bulb of Fasted Mice

**DOI:** 10.3390/genes11040433

**Published:** 2020-04-16

**Authors:** Jing Wu, Penglai Liu, Fengjiao Chen, Lingying Ge, Yifan Lu, Anan Li

**Affiliations:** 1 Jiangsu Key Laboratory of Brain Disease and Bioinformation, Research Center for Biochemistry and Molecular Biology, Xuzhou Medical University, Xuzhou 221004, China; jingwu@stu.xzhmu.edu.cn (J.W.); penglailiu@gmail.com (P.L.); fengjiaochen@xzhmu.edu.cn (F.C.); 2 The Second Clinical Medical College, Xuzhou Medical University, Xuzhou 221004, China; gelingying@outlook.com (L.G.); lyf00614@outlook.com (Y.L.)

**Keywords:** olfactory bulb, nutritional status, in vivo electrophysiological recording, odor representation

## Abstract

Olfaction and satiety status influence each other: cues from the olfactory system modulate eating behavior, and satiety affects olfactory abilities. However, the neural mechanisms governing the interactions between olfaction and satiety are unknown. Here, we investigate how an animal’s nutritional state modulates neural activity and odor representation in the mitral/tufted cells of the olfactory bulb, a key olfactory center that plays important roles in odor processing and representation. At the single-cell level, we found that the spontaneous firing rate of mitral/tufted cells and the number of cells showing an excitatory response both increased when mice were in a fasted state. However, the neural discrimination of odors slightly decreased. Although ongoing baseline and odor-evoked beta oscillations in the local field potential in the olfactory bulb were unchanged with fasting, the amplitude of odor-evoked gamma oscillations significantly decreased in a fasted state. These neural changes in the olfactory bulb were independent of the sniffing pattern, since both sniffing frequency and mean inhalation duration did not change with fasting. These results provide new information toward understanding the neural circuit mechanisms by which olfaction is modulated by nutritional status.

## 1. Introduction

Food intake is a complex process in which both homoeostatic regulation and hedonic sensations are critically involved. Most sensory systems influence food detection and consumption [1,2,3,4]. However, of all the sensory modalities, olfaction contributes the most to the hedonic evaluation of a food and its eventual possible consumption [1,2,5]. Conversely, metabolic states such as fasting or satiation have been reported to increase or decrease olfactory detection and discrimination in both humans and rodents [6,7,8]. Although it is well known that olfaction and satiety status influence each other, the underlying neural mechanisms are largely unknown. 

The representation of odor information is rather complex regarding the need to process parallel input from different olfactory receptors and trace amine-associated receptors expressed from more than 1000 genes in rodents [9,10,11]. The olfactory bulb (OB) is the first relay station and processing hub in the olfactory system. Recent studies have demonstrated that the OB plays a key role in the representation of odor identity, intensity, and timing [12,13,14,15]. In the OB, mitral/tufted cells (M/Ts) are the main output neurons that send the processed neural signals to higher olfactory centers for further information processing. Thus, factors that influence the activity of M/Ts or their ability to represent odors may cause deficiencies in olfactory function [12]. Therefore, it is important to decipher how nutritional status affects neural activity and odor representation in M/Ts.

Pioneering work performed by Pager found that more multiple units recorded from rat OB showed excitatory responses to a food odor when the animal was in a fasted state compared with a satiated state [16]. This finding was further supported by another study in which spikes were recorded from single units [17]. However, these studies only compared the number of units showing different response types; further in-depth analysis of how nutritional status influences neural discrimination of odors in M/Ts is lacking. Furthermore, whether the change in neural response in different nutritional states is dependent on changes in sniffing also remains unknown.

In the current study, we first tested how nutritional status modulates single-unit activity and neural discrimination of odors in M/Ts recorded from awake, head-fixed mice. We then investigated ongoing and odor-evoked local field potential (LFP) responses under different fasting states. Finally, we asked whether changes in the neural activity in the OB are related to changes in the animal’s sniffing pattern. We found that the excitability of neural activity is enhanced, but the neural discrimination of odors is slightly decreased in the OB of fasted mice.

## 2. Materials and Methods

### 2.1. Animals

Male eight-week-old C57BL/6J mice were used as experimental subjects and were housed under a 12 h light/dark cycle with food and water ad libitum. Normally, four or five mice were placed in one cage, but mice were housed individually after surgery for at least one week for recovery before further experiments. All experimental procedures complied with the animal care standards of the Xuzhou Medical University Institutional Animal Care and Use Committee (SYXK2015-0030).

### 2.2. Odorants and Preference/Avoidance Behavioral Test

**Figure 1:**
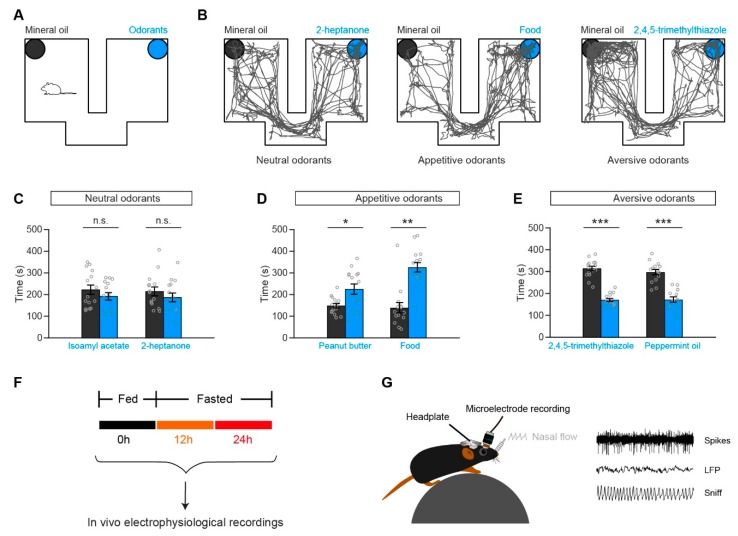
Paradigm for the preference/avoidance test and in vivo recordings. (**A**) Schematic of the preference/avoidance test. (**B**) Representative movement traces illustrating three different behaviors in C57BL/6J mice (left, a neutral odorant: 2-heptanone; middle, an appetitive odorant: food; right, an aversive odorant: 2,4,5-trimethylthiazole). (**C**–**E**) Quantification of time spent by mice in the chamber with the odorant (blue) versus mineral oil (black), for each type of odorant. n = 15 for each pair. (**C**) Mice showed neither preference nor avoidance for neutral odorants compared with mineral oil (paired *t*-tests: isoamyl acetate vs. mineral oil, *t*_(14)_ = −0.83, *p* = 0.42; 2-heptanone vs. mineral oil, *t*_(14)_ = 0.79, *p* = 0.44). (**D**) Mice showed preference for appetitive odorants compared with mineral oil (paired *t*-tests: peanut butter vs. mineral oil, *t*_(14)_ = 2.82, *p* = 0.013; Wilcoxon signed-rank test: Food vs. mineral oil, z = −2.78, *p* = 0.0053). (**E**) Mice showed avoidance for aversive odorants compared with mineral oil (paired *t*-tests: 2,4,5-trimethylthiazole vs. mineral oil, *t*_(14)_ = −8.58, *p* < 0.001; peppermint oil vs. mineral oil, *t*_(14)_ = −5.80, *p* < 0.001). (**F**) Diagram of the in vivo electrophysiological recordings. The recordings repeated at different metabolism states, including fasted for 0 h, 12 h, and 24 h. (**G**) Schematic representation of the methods for in vivo electrophysiological recordings in awake, head-fixed mice. n.s., not significant. * *p* < 0.05, ** *p* < 0.01, *** *p* < 0.001.

Odorants were applied in three groups: neutral odorants (isoamyl acetate, 2-heptanone), appetitive odorants (peanut butter, food pellets), and aversive odorants (2,4,5-trimethylthiazole, peppermint oil). The odorants were dissolved in mineral oil at 40% v/v dilution; peanut butter was used in their original states and food was dissolved in saline until the saline was saturated. For each animal, all six odors were tested. The interval between two odors was at least two days. The odors were presented in the order of appetitive odorants (Food, Peanut butter), neutral odorants (Isoamyl acetate, 2-heptanone), and aversive odorants (2,4,5-trimethylthiazole, peppermint oil). During testing, the odorant (50 μL), peanut butter (1 g), food (1 g), or mineral oil (50 μL) was placed on a filter paper in a dish (60 mm × 15 mm) and covered with cage bedding. A custom-designed test chamber (45 cm × 35 cm × 25 cm) with two equally sized compartments was used. Before the test, the mouse was placed into the chamber with empty dishes for 10 min to habituate to the environment. Then, preference for/avoidance of odorants was tested by exposing mice to the two compartments for 10 min, with an odorant in one compartment and mineral oil in the other compartment (the locations of the odorant and mineral oil were randomized) (Figure 1A). Odor preference or avoidance was reflected by the animal’s movement trace—the time spent in each chamber was calculated automatically by a computerized recording system.

### 2.3. Microelectrode Implantation

The microelectrodes (16-channel, Jiangsu Brain Medical Technology Co. Ltd, Nanjing, China) were implanted into a specific region of the brain as previously described [18,19]. Briefly, mice were anesthetized with pentobarbital sodium (90 mg/kg body weight, i.p.) and positioned in a stereotaxic frame. Eye ointment was applied to the eyes. The skull surface (from the midline of the orbits to the midpoint between the ears) was exposed, and a hole was drilled above the right OB for microelectrode implantation (anterior-posterior (AP): +4.0 mm; medial-lateral (ML): +1.0 mm). Then, the microelectrodes were positioned and lowered through the drilled holes until they reached the OB mitral cell layer at an average depth between 1.8 mm and 2.5 mm. A custom-designed head plate was attached to the skull with small screws and dental acrylic to enable head fixation during recordings. The body temperature of mice was maintained at 37 ± 0.5 °C throughout the surgery.

### 2.4. Spike and LFP Recordings

The recordings were initiated after the mice had recovered from surgery, as in previous studies [18,19,20,21]. Briefly, awake mice were head-fixed with two horizontal bars and were able to maneuver on an air-supported free-floating Styrofoam ball (Thinkerbiotech, Nanjing, China). For spike recordings, the signals from the microelectrodes were sent to a headstage, amplified by a 16-channel amplifier (Plexon DigiAmp (Plexon Inc, Dallas, TX, USA); bandpass filtered at 300–5000 Hz, 2000× gain), and sampled at 40 kHz by a Plexon Omniplex recording system. For LFP recordings, LFP signals were amplified (2000× gain, Plexon DigiAmp), filtered at 0.1–300 Hz, and sampled at 1 kHz. Spikes or LFP signals together with odor stimulation event markers were recorded via the same Plexon Omniplex recording system. The fasting started at 21:00 and finished at 21:00 the next day. Recordings were repeated on the same mice under different nutritional states: satiety, fasted for 12 h, and fasted for 24 h.

### 2.5. Odorant Presentation during Electrophysiological Recordings

The three sets of odorants described above were also used during electrophysiological recordings. Peanut butter was mixed with mineral oil at a 10% m/v dilution and food was dissolved in saline as described above. The other odorants were dissolved in mineral oil at 1% v/v dilution. During the odor delivery period, an odor delivery system (Thinkerbiotech, Nanjing, China) was used, as previously described [20,21]. There were 15 trials for each odorant. The six odorants were presented in a pseudo-randomized order, with no more than two successive presentations of the same odor. Each odor was delivered to the animal for 2 s with an inter-stimulus interval of 20 s. All six odorants were presented passively; the mice were not required to respond to the odors presented.

### 2.6. Measurement of Sniffing Parameters

The sniffing patterns of mice during electrophysiological recordings were recorded continuously by placing a cannula into one nasal cavity and connecting it to an airflow pressure sensor (Model No. 24PCEFA6G(EA), 0–0.5 psi, Honeywell) Surgical implantation of the nasal cannula was performed as described in previous studies [22,23]. The pressure transient signals were amplified (100× gain, Plexon DigiAmp) and sampled at 1 kHz by a Plexon Omniplex recording system. A sniff was defined as the point of transition from exhalation to inhalation.

### 2.7. Data Analysis

#### 2.7.1. Olfactory Preference/Avoidance Test

Preference time and avoidance time during the 10-min test were measured from the recorded videos using MATLAB. The test cage was divided into two compartments of equal area. Preference time was defined as the time spent in the compartment with a filter paper scented with peanut butter or food, and avoidance time was defined as the time spent in a compartment without a filter paper scented with 2,4,5-trimethylthiazole or peppermint oil [24].

**Figure 2:**
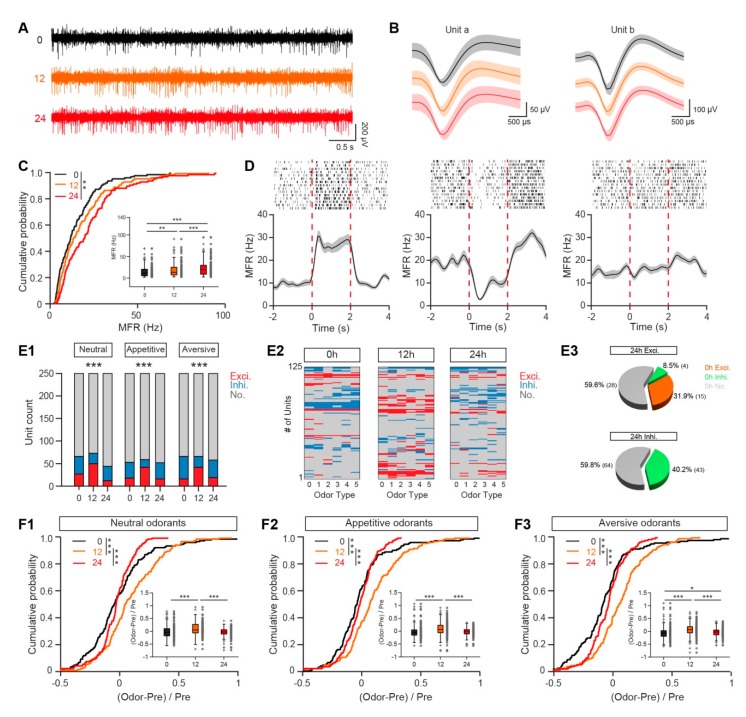
The firing of M/Ts recorded from the OB changes under different nutritional states. (**A**,**B**) Representative raw spike traces (**A**) and spike waveforms (**B**) recorded from a mouse fasted for 0 h (black), 12 h (orange), and 24 h (red) respectively. (**C**) Cumulative probability and box-and-whisker plot showing the spontaneous firing rate under different fasting states. Each gray circle represents the mean firing rate of a single unit. (Cumulative probability: Two-sample *K-S* test, 0 h vs. 12 h, *p* = 0.31, 0 h vs. 24 h, *p* = 0.0012, 12 h vs. 24 h, *p* = 0.073. Box-and-whisker plot: Friedman’s test, χ^2^_(2,248)_ = 65.10, *p* < 0.001, 0 h vs. 12 h, *p* = 0.0023, 0 h vs. 24 h, *p* < 0.001, 12 h vs. 24 h, *p* < 0.001). (**D**) Three examples of firing induced by isoamyl acetate when mice were in a satiated state. From left to right: an excitatory response, an inhibitory response, and no response. Top, raster plot. Bottom, peristimulus histograms for the firing rate, smoothed with a Gaussian filter with a standard deviation of 1500 ms. The red dotted lines indicate the period of odor stimulation. Error bars show the standard error of the mean (SEM). (**E**) The changes in the neural responses to odors under different fasting conditions. (**E1**). Stacked bar plots Neutral odorants (0 h, Excitory: 27/250, Inhibitory: 39/250, No response: 184/250; 12 h, Excitatory: 50/250, Inhibitory: 23/250, No response: 177/250; 24 h, Excitatory: 12/250, Inhibitory: 32/250, No response: 206/250). Chi-Square Tests: χ^2^_(4)_ = 31.23, *p* = 0.000003, Exci. 0 h vs. Exci. 12 h, *p* < 0.05, Exci. 0 h vs. Exci. 24 h, *p* < 0.05, Exci. 12 h vs. Exci. 24 h, *p* < 0.05. Appetitive odorants (0 h, Excitory: 18/250, Inhibitory: 35/250, No response: 197/250; 12 h, Excitory: 42/250, Inhibitory: 17/250, No response: 191/250; 24 h, Excitory: 16/250, Inhibitory: 36/250, No response: 198/250). Chi-Square Tests: χ^2^_(4)_ = 24.47, *p* = 0.000064, Exci. 0 h vs. Exci. 12 h, *p* < 0.05, Exci. 12 h vs. Exci. 24 h, *p* < 0.05. Aversive odorants (0 h, Excitory: 16/250, Inhibitory: 50/250, No response: 184/250; 12 h, Excitory: 42/250, Inhibitory: 24/250, No response: 184/250; 24 h, Excitory: 19/250, Inhibitory: 39/250, No response: 192/250). Chi-Square Tests: χ^2^_(4)_ = 25.04, *p* = 0.000049, Exci. 0 h vs. Exci. 12 h, *p* < 0.05, Exci. 12 h vs. Exci. 24 h, *p* < 0.05. (**E2**). Pseudocolor plots represent odor-evoked responses for each unit at different metabolism states, including fasted for 0 h, 12 h, and 24 h. (**E3**). Among the number of unit-odor pairs with excitatory response (Top) or inhibitory response (Bottom) at fasted for 24 h, the percentages for unit-odor pairs showing excitatory (orange), inhibitory (green) or no response (gray) at fasted for 0 h. (**F**) Cumulative probability and box-and-whisker plots of the odor-evoked firing rate. (**F1**). Cumulative probability: Two-sample *K-S* test: 0 h vs. 12 h, *p* < 0.001, 0 h vs. 24 h, *p* = 0.082, 12 h vs. 24 h, *p* < 0.001. Box-and-whisker plot: Friedman’s test: χ^2^_(2,310)_ = 35.81, *p* < 0.001, 0 h vs. 12 h, *p* < 0.001, 12 h vs. 24 h, *p* < 0.001. (**F2**). Cumulative probability: Two-sample *K-S* test: 0 h vs. 12 h, *p* < 0.001, 0 h vs. 24 h, *p* = 0.24, 12 h vs. 24 h, *p* < 0.001. Box-and-whisker plot: Friedman’s test: χ^2^_(2,310)_ = 17.78, *p* = 0.00014, 0 h vs. 12 h, *p* = 0.00085, 12 h vs. 24 h, *p* = 0.00068. (**F3**). Cumulative probability: Two-sample *K-S* test: 0 h vs. 12 h, *p* < 0.001, 0 h vs. 24 h, *p* = 0.023, 12 h vs. 24 h, *p* < 0.001. Box-and-whisker plot: Friedman’s test: χ^2^_(2,310)_ = 30.36, *p* < 0.001, 0 h vs. 12 h, *p* < 0.001, 12 h vs. 24 h, *p* < 0.001. * *p* < 0.05, ** *p* < 0.01, *** *p* < 0.001.

Offline spike sorting and statistics of single-cell spiking data: When we did spike sorting, all the files were put together to make sure signals recorded at different stages were from same units. Similar to previous studies, single units were sorted and identified with principal components analysis in Offline Sorter V4 software [18,19]. In addition, we performed further analysis on all recorded unit to double check that the spikes were from the same units (repeated measures ANOVA on the spike amplitude and half-width). To generate the peristimulus time histogram (PSTH), spikes 2 s before and 4 s after the onset of odor stimulation were extracted for each trial and the spike firing rate was averaged over 100-ms bins (Figure 2E). The spontaneous firing rate (during the 2 s before odor stimulation) and the odor-evoked firing rate (during the 2 s after odor stimulation) were calculated by averaging the frequency of spikes during these 2 s periods. To test whether an odor evoked a significant response, we used a paired *t*-test to compare the baseline firing rate with the odor-evoked firing rate across all the trials for each cell–odor pair. If the *p* value was >0.05, the cell–odor pair was defined as nonresponsive. If the *p* value was <0.05, the cell–odor pair was defined as responsive and was further categorized as excitatory (if the odor-evoked firing rate was higher than the baseline firing rate) or inhibitory (if the odor-evoked firing rate was lower than the baseline firing rate).

#### 2.7.2. Analysis of LFP Signals

Programs written in MATLAB were used to analyze the LFP signals. Raw data 2 s prior to the onset of odor stimulation were used to represent the ongoing baseline LFP activity. A time–frequency transformation was performed on this 2-s window. For odor-evoked responses, the data 2 s prior to and 4 s after the onset of odor stimulation were selected for presentation and further analysis. Similar to previous studies [20,21], we divided the LFP signals into four frequency bands: theta (2–12 Hz), beta (15–35 Hz), low gamma (36–65 Hz), and high gamma (66–95 Hz). However, we focused only on the beta and high gamma bands in our analysis since odors usually evoke strong and reliable responses within these two frequency bands. Spectral power was computed using MATLAB’s STFT method (The MathWorks). For each trial, the baseline was normalized to 1, and all the trials for each odor were averaged for further analysis.

#### 2.7.3. Receiver Operating Characteristic (ROC) Analysis

Receiver operating characteristics (ROCs) were used to assess the classification of responses evoked by odor pairs, and were estimated using the roc function in MATLAB. Mean firing rate during odor stimuli with a 2-s bin was utilized in ROC analysis. The area under the ROC (auROC) is a nonparametric measure of the discriminability of two distributions. We used the auROC to assess the classification of the two odors within an odor pair. An auROC curve is defined from 0.5 to 1.0. A value of 0.5 indicates completely overlapping distributions, whereas a value of 1 predicts perfect discriminability [19].

#### 2.7.4. Statistics

Data were analyzed in MATLAB. The Gaussian distribution of the data was assessed using the Anderson–Darling test. If the data sets were normally distributed, the data were tested for significance using a paired *t*-test (two related samples) or one-way repeated measures ANOVA (>2 related samples). If the data sets were non-normally distributed, the data were tested for significance using the Wilcoxon signed-rank test (two related samples), Friedman’s test (>2 related samples), or the Kruskal-Wallis test (>2 unrelated samples). Tukey post-hoc tests were used to directly assess group differences following ANOVA where appropriate. Where boxplots are used to represent the data, the median is plotted as a line within a box formed by the 25th (q_1_) and 75th (q_3_) percentiles. Points are drawn as outliers if they are larger than q_3_ + w × (q_3_ − q_1_) or smaller than q_1_ − w × (q_3_ − q_1_). 

## 3. Results

To test the odor responses of OB neurons under satiated and fasted states, different types of odorants were used. Isoamyl acetate and 2-heptanone were used as neutral odorants, peanut butter [24,25,26] and food odor were used as appetitive odorants, and 2,4,5-trimethylthiazole and peppermint oil [27] were used as aversive odorants. To confirm that the mice had a preference for the appetitive odorants and avoided the aversive odorants, we performed a preference/avoidance test (Figure 1A). An example is illustrated in Figure 1B: although the mouse had no preference or avoidance for 2-heptanone versus mineral oil, it demonstrated a preference for food odor and avoidance of 2,4,5-trimethylthiazole. Further analysis across all the mice tested demonstrated that isoamyl acetate/2-heptanone, peanut butter/food odor, and 2,4,5-trimethylthiazole/peppermint oil were neutral, appetitive, and aversive odorants for mice, respectively (Figure 1C–E). 

To compare the neural activity and sniffing patterns in satiated and fasted states, signals were recorded before the removal of food, and 12 hours and 24 hours after the removal of food (Figure 1F). Sniffing signals and neural activity, including spikes and LFP, were recorded simultaneously in awake, head-fixed mice (Figure 1G).

### 3.1. Baseline Firing Rate and Odor-Evoked Responses are Both Enhanced in a Fasted State

First, we investigated the spontaneous neural activity of single M/Ts under different nutritional states. Extracellular microelectrodes were placed into the mitral cell layer and single M/T units were isolated and sorted as described previously [18,19,23]. As in previous studies, we observed strong spontaneous firing of M/Ts in awake mice (Figure 2A). Figure 2B shows examples of two single M/Ts sorted from microelectrode recordings. The shapes of these units were similar across satiated and fasted states, indicating that the signals were likely collected from the same units under different states. We performed further analysis on all recorded unit to double check that the spikes were from the same units (repeated measures ANOVA on the spike amplitude and half-width). The data showed that signals recorded at different stages were not significantly different (Friedman’s test, for amplitude, x^2^_(2,248)_ = 4.501, *p* = 0.11; for half-width, x^2^_(2,248)_ = 4.36, *p* = 0.11), indicating they were likely from same units. Compared with the satiated state, the spontaneous firing of M/Ts was significantly increased at 12 hours after removal of food, and further increased at 24 hours after removal of food (Figure 2C). In addition, we also performed control experiment in which we recorded data at different time points (0 h, 12 h, 24 h), but the food was not removed. The data showed that spontaneous firing of M/Ts at different time point with food were not significantly different (Two-sample *K-S* test, 0 h vs. 12 h, *p* = 0.18, 0 h vs. 24 h, *p* = 0.10, 12 h vs. 24 h, *p* = 0.99; Friedman’s test, x^2^_(2,248)_ = 0.4, *p* = 0.82).

Next, we investigated how fasting affects the odor-evoked responses of M/Ts. Consistent with the findings from previous studies, M/Ts showed both excitatory and inhibitory responses to odor stimulation in the satiated state (Figure 2D). Compared with the satiated state, the number of units showing excitatory responses was significantly increased 12 h after the removal of food and the number of units showing inhibitory responses was significantly decreased, for all three types of odorant (neutral, appetitive, and aversive) (Figure 2(E1)). Interestingly, this tendency was not observed 24 h after the removal of food (Figure 2(E1)). Control experiment showed that excitatory responses at different time point with food was not significantly different, for all three types of odorant (Chi-Square Tests, all *p* > 0.05). Since the number of responsive units under satiated state was similar with 24 h after the removal of food, this raises the question that whether they were the same set of units. We provided further presentations of the odor-evoked responses (Figure 2(E2,E3)). Interestingly, we found that most of the units showing excitatory responses under over-fasted state (24 h after removal of food) were not the same units showing excitatory responses under satiated state, indicating that the neural connectivity was reconfigured under over-fasted state (Figure 2(E2,E3)).

To further compare the amplitude of odor-evoked responses under different states, we analyzed the normalized odor response. We found that, compared with the satiated state, the odor response was increased 12 h after the removal of food for all odorants tested, and recovered 24 h after the removal of food (Figure 2F). This finding is consistent with the changes in the number of responsive units under different fasting states. Together, these results from single unit recordings indicate that the excitability of M/Ts is enhanced in fasted mice.

### 3.2. Neural Discrimination of Odors is Slightly Decreased in the OB of Fasted Mice

**Figure 3:**
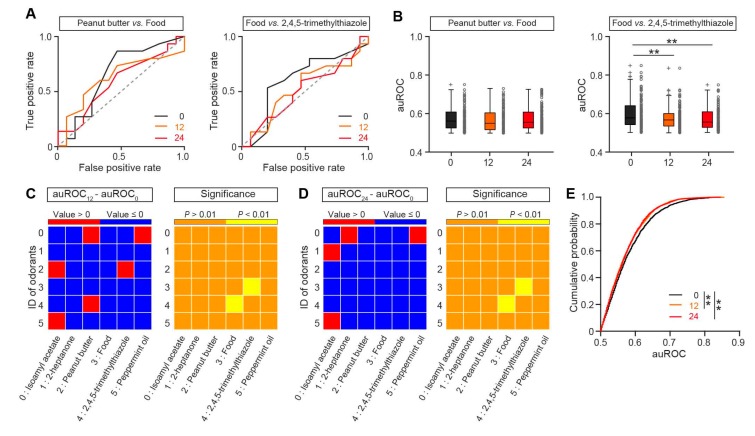
Nutritional status influence odor representation in M/Ts. (**A**) Example receiver operating characteristic (ROC) plots of the neural responses to peanut butter vs. food (left) and food vs. 2,4,5-trimethylthiazol (right) when mice had been fasted for 0 h (black), 12 h (orange), or 24 h (red). (**B**) Comparison of areas under the ROC (auROCs) for the neural responses to peanut butter vs. food (left) and food vs. 2,4,5-trimethylthiazol (right). Each gray circle represents the auROC value for a single unit. Left. Friedman’s test: χ^2^_(2,248)_ = 0.36, *p* = 0.83. Right. Friedman’s test: χ^2^_(2,248)_ = 14.06, *p* = 0.00089, 0 h vs. 12 h, *p* = 0.0059, 0 h vs. 24 h, *p* = 0.0020. (**C**,**D**) Pseudocolor plots of the D-value and the *p* value when auROC12 was compared with auROC0 (**C**) or when auROC24 was compared with auROC0 (**D**). (**E**) Cumulative probability of auROCs. Two-sample *K-S* test: 0 h vs. 12 h, *p* = 0.0092, 0 h vs. 24 h, *p* = 0.0024, 12 h vs. 24 h, *p* = 0.90. ** *p* < 0.01.

The significant difference in the excitability of M/Ts in satiated and fasted states raises the question of whether odor discrimination by single M/Ts is different under these two states. To investigate this, we characterized the ability of single-unit M/Ts to discriminate the odors. To compare the classification of odor-evoked responses under different nutritional states, we calculated the receiver operating characteristics (ROC) [19,28]. Figure 3A shows two example ROC plots—whereas the ROC curves were similar under different states for the odor pair of peanut butter and food odor (Figure 3A, left), the ROC curves were different under different states for the odor pair of food odor and 2,4,5-trimethylthiazole (Figure 3A, right). ROC analysis of all animals showed that the auROC values for the peanut butter/food odor pair were similar for satiated and fasted states (Figure 3B, left) but the auROC values for the food odor/2,4,5-trimethylthiazole pair were significantly different in different states (Figure 3B, right). We analyzed all odor pairs and found that most of the odor pairs showed smaller auROC values under fasting (Figure 3C,D, left), although no specific odor pair other than food odor/2,4,5-trimethylthiazole had a significant difference in auROC values between the satiated and fasted states (Figure 3C,D, right). The cumulative probability of auROCs analysis of all odor pairs from all animals showed that the auROC values under the satiated state were significantly larger than under the fasted states (Figure 3E) both 12 h and 24 h after the removal of food. Thus, these results indicate that, compared with the satiated state, there was a tendency that the neural discrimination was slightly decreased in the OB of fasted mice under awake, head-fixed conditions.

### 3.3. Odor-Evoked Gamma Responses are Decreased in a Fasted State

**Figure 4:**
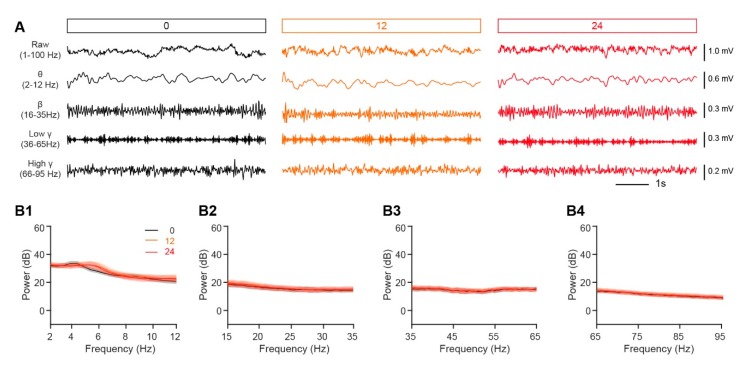
Nutritional status has no significant effect on the ongoing LFP in the OB. (**A**) Examples of ongoing baseline LFP signals from a single mouse fasted for 0 h (black), 12 h (orange), and 24 h (red). The first row shows 6 s of the raw trace; the second to fifth rows show the filtered signal (theta, beta, low gamma, and high gamma, respectively). (**B**) The averaged power spectrum of the ongoing LFP signals. (**B1**–**B4**) show the averaged power spectrum in the theta (**B1**), beta (**B2**), low gamma (**B3**), and high gamma (**B4**) bands across the group of mice. (**B1**). Friedman’s test: χ^2^ (2,38) = 0.57, *p* = 0.75. (**B2**). Friedman’s test: χ^2^ (2, 80) = 2.06, *p* = 0.36. (**B3**). Friedman’s test: χ^2^ (2, 118) = 2.85, *p* = 0.24. (**B4**). One-way rANOVA: *F* (2,116) = 1.09, *p* = 0.34. Error bars show the SEM.

Whereas single-unit spiking reflects the activity in a single M/T cell, oscillations in the LFP reflect the neural activity in the population of cells surrounding the recording site [29]. Oscillations recorded from the OB contain important information relating to the chemical properties of odors, olfactory learning, and odor discrimination [30,31]. Thus, we next compared the LFP signals in awake, head-fixed mice in satiated and fasted states. As in previous studies, the raw LFP signals were divided into different frequency bands: theta, 2–12 Hz; beta, 15–35 Hz; low gamma, 36–65 Hz; and high gamma, 66–95 Hz (Figure 4A). No significant differences were found between the satiated and fasted states for any frequency band of the ongoing, baseline LFP (Figure 4B). 

**Figure 5:**
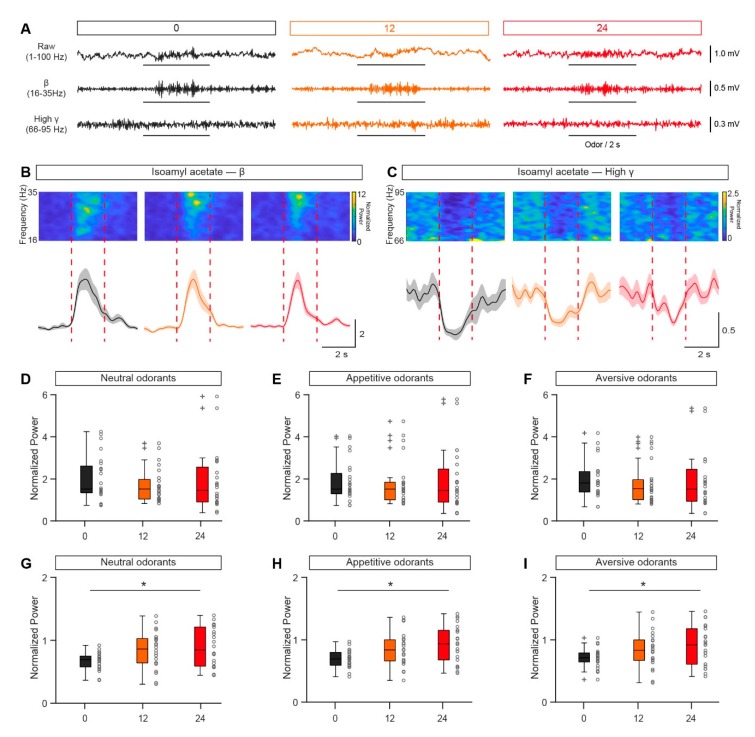
Nutritional status modulates odor-evoked LFP responses. (**A**) Responses of the raw LFP trace and the filtered beta and high gamma bands to odor stimulation under different nutritional states. Black bars indicate odor stimulation. (**B**,**C**) Top: Example power spectra for odor-evoked beta (**B**) and high gamma oscillations in the OB when mice were fasted for 0 h (black), 12 h (orange), or 24 h (red). Bottom: Trial-averaged normalized traces of odor-evoked beta (**B**) and high gamma (**C**) responses. The red dotted lines indicate the period of odor stimulation. Error bars show the SEM. (**D**–**F**) Comparison of the power in the normalized odor-evoked beta band evoked by neutral (**D**), appetitive (**E**), or aversive odorants (**F**) under different fasting states (n = 12, neutral odorants, Friedman’s test: χ^2^_(2,46)_ = 1, *p* = 0.61; appetitive odorants, Friedman’s test: χ^2^_(2,46)_ = 0.58, *p* = 0.75; aversive odorants, Friedman’s test: χ^2^_(2,46)_ = 4.08, *p* = 0.13). (**G**–**I**) Comparison of the power in the normalized odor-evoked high gamma evoked by neutral (**G**), appetitive (**H**), or aversive odorants (**I**) under different fasting conditions (n = 12, neutral odorants, Friedman’s test: χ^2^_(2,42)_ = 8.27, *p* = 0.016, 0 h vs. 24 h, *p* = 0.012; appetitive odorants, Friedman’s test: χ^2^_(2,42)_= 7.64, *p* = 0.022, 0 h vs. 24 h, *p* = 0.018; aversive odorants, Friedman’s test: χ^2^_(2,42)_ = 8.45, *p* = 0.015, 0 h vs. 24 h, *p* = 0.018). * *p* < 0.05.

Next, we investigated whether odor-evoked LFP responses differed with fasting state in awake, head-fixed mice. Figure 5A–C show the LFP response to isoamyl acetate in a single mouse across different fasting states. In all states, there was a strong beta response to the odor and a high gamma response. However, although the amplitude of the isoamyl acetate-induced beta-band response was similar in the fasted state and the satiated state, the amplitude of the isoamyl acetate-induced high-gamma response decreased with fasting (Figure 5B,C). This phenomenon was also observed for other odorants (e.g., Figure 5D–I). 

When the odorant-induced changes in LFP power under satiated and fasted states were surveyed across all mice and odors (Figure 5D–I), we found that there was no significant difference between satiated and fasted states for the odor-evoked beta response (Figure 5D–F), but the high gamma response was significantly reduced for all the three types of odorants with the longest fasting duration (48 h after the removal of food, Figure 5G–I). These results indicate that odor-evoked inhibition of the neural network in the OB is reduced in a fasted state in awake, head-fixed mice.

### 3.4. Slight Decrease in the Sniffing Volume Between Satiated and Fasted States

**Figure 6:**
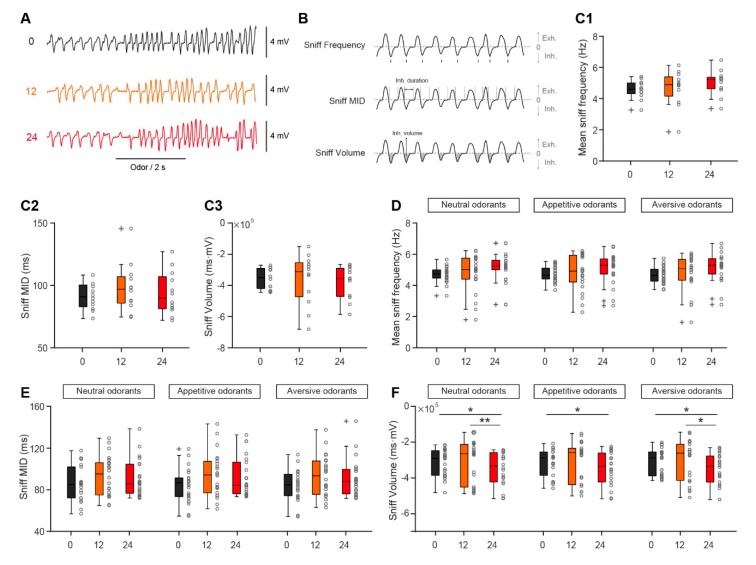
Change in the sniffing volume under different nutritional states. (**A**) Raw sniff trace recorded from a representative mouse fasted for 0 h (black), 12 h (orange), or 24 h (red). (**B**) Diagram illustrating the extraction of sniff frequency, mean inhalation duration (MID), and volume from a sample nasal flow trace. (**C**) Sniffing recorded under baseline conditions (no odor presentation) after fasting for different durations. (**C1**). Mean sniff frequency. One-way ANOVA: *F*_(2,22)_ = 1.30, *p* = 0.29. (**C2**). Sniff mean inhalation duration (MID). Friedman’s test: χ^2^_(2,22)_ = 2.17, *p* = 0.34. (**C3**). Sniff volume. Friedman’s test: χ^2^_(2,22)_ = 3.17, *p* = 0.21. (**D**) Odor-evoked mean sniff frequency recorded when mice were fasted for 0 h (black), 12 h (orange), or 24 h (red). Neutral odorants: Friedman’s test: χ^2^_(2,42)_ = 3.91, *p* = 0.14. Appetitive odorants: Friedman’s test: χ^2^_(2,42)_ = 3.27, *p* = 0.19. Aversive odorants: Friedman’s test: χ^2^_(2,42)_ = 4.73, *p* = 0.094. (**E**) Fasting has no effect on odor-evoked sniff MID. Neutral odorants: Friedman’s test: χ^2^_(2,42)_ = 1.91, *p* = 0.38. Appetitive odorants: Friedman’s test: χ^2^_(2,42)_ = 2.45, *p* = 0.29. Aversive odorants: Friedman’s test: χ^2^_(2,42)_ = 1.91, *p* = 0.38. (**F**) Odor-evoked sniff volume changes with fasting. Neutral odorants: Friedman’s test: χ^2^_(2,42)_ = 10.18, *p* = 0.0062, 0 h vs. 24 h, *p* = 0.042, 12 h vs. 24 h, *p* = 0.0072. Appetitive odorants: Friedman’s test: χ^2^_(2,42)_ = 6.91, *p* = 0.032, 0 h vs. 24 h, *p* = 0.042. Aversive odorants: Friedman’s test: χ^2^_(2,42)_ = 9.91, *p* = 0.0071, 0 h vs. 24 h, *p* = 0.028, 12 h vs. 24 h, *p* = 0.012. * *p* < 0.05, ** *p* < 0.01.

Since mice rely on respiration/sniffing to sample odors, sniffing plays an important role in odor processing and representation in the OB [32]. Thus, we next investigated whether sniffing changes under satiated and fasted states. The raw sniffing patterns recorded under different states are shown in Figure 6A. We analyzed different aspects of the sniffing signal, including sniffing frequency, mean inhalation duration (MID), and volume (Figure 6B). As shown in Figure 6C, there were no significant changes in ongoing sniffing frequency, MID, or volume in the fasted state versus the satiated state. Analysis of the odor-evoked sniffing data across all animals supports the result that only sniffing volume changed with fasting (24 h after the removal of food); this phenomenon was observed consistently for all three types of odorant (Figure 6D–F). Thus, taken together, these results indicate that there is only a weak difference in the sniffing pattern between satiated and fasted states, limited to a slight decrease in sniffing volume.

## 4. Discussion

In this study, we investigated how nutritional states modulate the neural activity and neural representation of odors in the M/Ts of awake, head-fixed mice. Our data indicate that, in a fasted state, both the spontaneous firing rate of M/Ts and the number of cells showing an excitatory response to odors are increased at the single-cell level, suggesting that the excitability of neural activity is enhanced. Unexpectedly, the ability of M/Ts to discriminate different odors is slightly decreased in the fasted state, indicating that the effect of different nutritional states on olfaction is complex and that higher brain centers beyond the OB are likely involved.

Many previous studies have investigated behavioral odor detection and discrimination under different nutritional states in humans and rodents [2,5]. Fasting results in an increased perception of some food-related odors in humans [33] and olfactory sensitivity to a neutral odor increases in fasted rats [7]. The neural mechanisms underlying these behavioral observations are not clear, even though many studies have focused on this issue and a lot of data have been collected [16,17,21,34,35,36,37]. Since the OB is the first olfactory center and plays critical roles in odor information processing, most of the studies of different nutritional states examined neural activity in the OB [2,16,17,18,34,37]. In the 1970s, Pager found that the electrical activity of mitral cells was dependent on nutritional status: fasting selectively increased mitral cell multiunit responses to food odor [16]. This finding was partially supported by another study in which mitral cell single-unit responses to odor increased with fasting, regardless of the odorant [17]. Our results are consistent with the latter since we found that both the number of M/Ts showing an excitatory response and the amplitude of the response were greater in a fasted state, and that these increments were independent of odor type. In addition, we found that the baseline firing rate of single M/Ts was significantly increased in a fasted state. Therefore, the excitability of M/T neural activity is enhanced in the OB of fasted mice. 

While the spikes from single unit in the OB have the capacity to encode odor identity in awake behaving rodents [13,22], LFP recorded form the OB reflect temporally coordinated neuronal ensembles and provides a reference for spike timing-based codes [38]. In the OB, the spikes from M/Ts are highly correlated with high-gamma oscillation and their correlation carries important information on odor identity [22,39]. Thus, it is not surprising that the increase in excitatory neural activity in the OB in a fasted state is further supported by our LFP recordings. Although the ongoing baseline LFP activity did not change from the satiated to the fasted state, the amplitude of the odor-evoked gamma oscillations was significantly decreased, indicating an increase in an excitatory component of the LFP or a decrease in an inhibitory component during odor stimulation in a fasted state. Since gamma oscillations in the OB are generated by the an interaction between M/Ts and granule cells [39,40], and since the firing rate of M/Ts is greater in the fasted state, the decrease in the odor response in the fasted state is likely due to the increased excitability of the M/Ts. Whether the firing of granule cells changes with fasting is an interesting question; cell-type-specific recording of the spikes from granule cells will be needed to address this, using, for example, the juxtacellular “loose-patch” recording method [41]. 

Although previous study performed in free-moving rats found that odor-evoked beta response was affected by fasting [34], we did not find significant changes after fasting during passive odor stimulation in present study. However, it is likely that fasting would change odor-evoked beta oscillation if the mice detect the odors actively, e.g., during the odor discrimination task, since beta oscillation is critically involved in the learning process [39,42]. Previous studies have demonstrated that beta oscillation is generated by the interaction between the OB and piriform cortex [43], and the centrifugal input from the piriform cortex to the OB should be critical for the beta oscillation. Strikingly, CB1 receptors are expressed in these centrifugal fibers and they play important roles in food intake [1]. Thus, odor-evoked beta oscillation is likely modulated by fasting during active rather than passive odor sampling.

Another important finding in our study is that the neural discrimination of odors by M/Ts is slightly decreased in the fasted state. Interestingly, for individual odor pairs, a significant decrease in odor discrimination was found only for food odor and 2,4,5-trimethylthiazole (Figure 3). Since behaviorally, odor discrimination is better during a fasted state [7,33], this finding was unexpected. One possible explanation is that the mice in our experiment were head-fixed when the odor was delivered rather than free-moving. The experiment was performed with head fixation since odor sampling is more stable with head fixation and more dynamic when mice are free moving [12]. Although the behavioral output of the animals is generally similar under the two conditions [44], the neural activity may be very different because of the different status. Another possible explanation for the unexpected decrease in neural discrimination of odors is that the mice in our study may have been over-fasted, since we observed that the sniffing volume decreased slightly under a fasted state. Thus, the decrease in odor discrimination by single M/Ts in a fasted state is likely due to changes in the animals’ general state, such as the metabolic condition. Furthermore, we used the firing rate to calculate odor representation in the present study; however, temporal information can also be used for odor representation in the OB [13,14,45]. Finally, the OB receives dense modulation from higher brain areas, including both feedback and centrifugal inputs, and all of these projections dramatically modulate cell activity, odor information processing, and odor representation in the OB [12,46,47,48]. The change in odor discrimination at the single cell level observed in the OB is the result of complex network interactions between the OB and other higher brain centers. Thus, the neural representation in the OB may not necessarily represent the final behavioral output. 

In the OB, activity in the circuits and in the different neuronal subtypes is modulated dramatically by sniffing [32]. The changes in M/T neural activity and odor discrimination in the fasted state observed in the present study may be due to changes in the sniffing pattern. However, we found only a slight decrease in sniffing volume with fasting; both sniffing frequency and MID remained unchanged. This indicates that the modulation of neural activity in the OB by nutritional state is independent of sniffing. Interestingly, a higher sniffing frequency has been reported in fasted free-moving rats [49], but we did not observe a significant change in sniffing frequency in head-fixed mice. This discrepancy is likely due to differences between the free-moving and head-fixed states. In free-moving state, animals are under active condition and they can move around and locate potential food source by fast sniffing. However, in head-fixed state, animals are under passive condition, cannot not move, and thus have no enthusiasms to find food and need not sniff fast. Thus, this discrepancy between the two studies is likely due to differences between the free-moving and head-fixed states. Future studies could test this possibility by monitoring sniffing signals in the same animals under free-moving and head-fixed conditions. 

In summary, we found that the excitability of neural activity is enhanced but neural discrimination of odors is slightly decreased in the OB of fasted mice under awake, head-fixed conditions. Although a more detailed investigation into the underlying neural mechanisms is warranted, our results represent a first step toward understanding the neural circuit mechanisms by which olfaction is modulated by nutritional status.
